# Validation of work pressure and associated factors influencing hospital nurse turnover: a cross-sectional investigation in Shaanxi Province, China

**DOI:** 10.1186/s12913-017-2056-z

**Published:** 2017-02-03

**Authors:** Huiyun Yang, Jingwen Lv, Xi Zhou, Huitong Liu, Baibing Mi

**Affiliations:** 1 The Second Affiliated Hospital of Xi’an Jiaotong University, Xi’an, Shaanxi China; 2 Shaanxi Provincial People’s Hospital, Xi’an, Shaanxi China; 3 Department of Epidemiology and Biostatistics, School of Public Health, Xi’an Jiaotong University Health Science Center, Xi’an, Shaanxi China

**Keywords:** Nurses, Turnover intention, Work stress, Midwest China

## Abstract

**Background:**

Nurses' turnover is a major contributor to nursing shortages, strongly influenced by nurses’ intentions to leave. Several factors influencing the turnover intention have been well identified in Western countries and large cities in China. However, whether these factors also contribute to nurses' work stress in Midwest China are still unclear. The main purpose of this study was to examine the work pressure and associated factors influencing the nurses’ intent to leave.

**Methods:**

A cross-sectional questionnaire-based survey with multistage sampling was conducted by recruiting 800 employed registered nurses with >1 year of work experience. Chi-square test and multi-factor logistic regression were applied to attain the relative comparisons. Sub-group analysis was conducted to explore the different turnover intention patterns in different age groups.

**Results:**

The turnover intention was classified as strong/very-strong (19%), weak (62%), and very-weak (19%). Among the factors influencing the nurses’ desire to leave the profession, work pressure was the most prominent. The predominantly associated factors contributing the work stress were age, experience, and workload. However, the scale of income did not affect the intent to leave decision. Pediatrics was identified to be the highest tormented department with a significant (*P* < 0.05) turnover of nurses. Among different age sub-groups, 30–39 age group nurses in Secondary hospitals demonstrate a stronger intent to leave.

**Conclusion:**

Nurses’ turnover intentions were associated with stress, age, job duty, and career commitment in Shaanxi Province. The intent to leave is dynamically multifactorial, and effective managements and supportive strategies are needed to reduce the nurses work stress accordingly.

## Background

For several years, nursing shortages have been a universally growing concern. In a 2002 survey conducted across nursing unions worldwide, 90 of the 105 organizations (86%) located in over 69 geographically distinct areas, reported nursing shortages in their jurisdiction [1]. In United States, it is estimated that the demand for registered nurses rises by 2–3% yearly, and that by the year 2025, there will be a shortage of 500,000 registered nurses [2]. The shortage of nurses was gradually accounted of in China, and the relationship between shortage of nurses and burnout intention was also more and more concerned [3–5].

The most recognized cause for nursing shortage appears to be attrition from nurses leaving the profession [2, 5]. Nursing turnover affects registered nurses as well as new graduates, which makes it costly for both society and healthcare organizations [6]. Nursing training requires both significant time and capital investments. In most industrialized countries, obtaining a nursing degree requires 3 to 5 years of professional education, examinations and clinical internships or residency [7]. Nonetheless, turnover intentions in new graduates are high [8]. For organization, the cost of both early and late turnover from the profession is high. Reducing resignation rates, by helping nurses to remain on the job, is an appealing strategy to reduce turnover and effectively address nursing shortages.

Turnover intention, a psychological disposition to leave an organization or a position, can serve as an excellent predictor of resignation among nurses [9–13]. Many factors have been associated with turnover intention. Factors that are work related include high job demand, perceived autonomy at work, support from superior or peers, and job satisfaction [14]. Some factors are associated with personal characteristics and aspirations, such as professional self-image, resilience and work-life interference [15–17].

Investigating the factors associated with turnover intention can help hospital administrators to take preventive measures against nursing turnover [18] and eliminate potential problems in clinical nursing services that might lead to nurses resignation [19–21]. Moreover, several conceptual models are developed on the basis of previous reports; the consensus on the actual factors reasoning the intent to leave is limited. An exhaustive analysis by Gilmartin identifies a gap in the conceptualization of work setting from the general nursing management compared to other profession [22]. Gilmartin argued a stronger integration of theoretical approaches across the field towards a better understanding of the dynamic nature of nurses’ voluntary turnover [22]. In addition, the mechanistic failures are attributed to the buoyancy in the contributing factors amassed by the cultural differences among the developed and developing nations. Therefore, reaching a consensus on conceptual model requires additional data and further validation owing to the volatility in the geographical settings, hospital size, staffing and general advancements in the life-style.

**Fig. 1:**
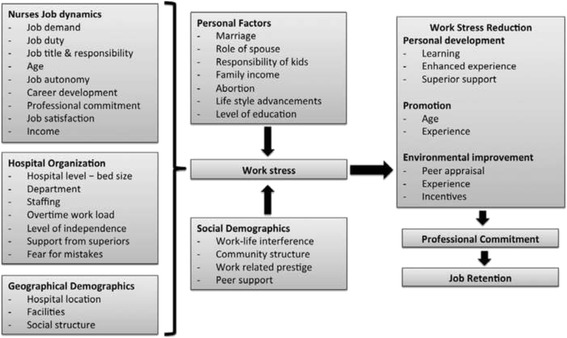
Hypothesized relationship between different factors and nurse's intension to leave

Until now, few large sample studies on the predictive factors associated with nurses’ turnover intentions and resignation factors have been conducted in Midwest China. Although work stress and associated factors have emerged as prime factors affecting the intent to leave, these are subject to further validation. Shaanxi province is located in the western region of China; the 2014 statistics shows 977 hospitals with 97,221 registered nurses catering an approximate population of 37 million [23]. Therefore, the nurses’ turnover imposes severe constrains on the healthcare system. Hence, the variability in the currently known factors influencing the intent to leave must be identified and accounted for the appropriate managerial policy considerations. In our research, we conducted an investigation among nurses in Shaanxi Province and aimed to analyze the status of 721 nurse’s intentions to leave the profession, as well as factors that influence these intentions. A conceptual framework is drafted to explain the impact of job dynamics, hospital organization and geographic demographics on nurse's intention to leave (Fig. 1).

## Methods

### Study design

This is a cross-sectional survey, using multistage proportional sampling, of 800 licensed nurses who were currently working at a participating hospital (The Second Affiliated Hospital of Xi’an, Jiaotong University, Yan’an University Affiliated Hospital, Ankang City Central Hospital, Luochuan County Hospital, Xunyang County Hospital, and Ziyang County Hospital). Employed licensed nurses were recruited using a stratified sampling strategy. The province was divided into three regions (northern, middle and southern) and two hospitals from each region were randomly selected. First, the number of samples required from each hospital was calculated from the ratios of the number of nurses in each hospital and the total number of nurses in all 6 hospitals. Then, the number of samples required from each department within a hospital was calculated using the ratios of the number of nurses in each department and the total number of nurses in each hospital Finally, random sampling was performed in each surveyed department.

Registered clinical nurses who had been employed for ≥1 1 year were invited to participation. Nurses who were not currently working when the investigation was conducted, such as those on maternity or sick leave, nursing students in training or on refresher courses, and those who were not permanently employed by the hospital were excluded, as well as those who were training in a different hospital at the time of the investigation.

### Data collection

This study was conducted in 2013, by self-administered questionnaires. Quota sampling was carried out at each hospital with the nursing department director’s approval and signed consents were obtained from all participants. Then questionnaire survey was carried out on the nurses and was recycled on the spot.

### Validity and reliability of instruments

Seven standardized psychometric scales, previously used in domestic researches, were used to build the questionnaire. The *Questionnaire Form for Nurses’ General Information* was used to collect sociodemographic and professional information, such as the professional titles and job responsibilities. The *Chinese Nurses’ Practice Environment Scale* (C-NPES) is a 31-items scale evaluating the administrator’s management ability and leadership, development of the nursing profession, nursing quality, adequacy of nursing staff and medical records, and involvement in hospital affairs [24]. The items are scored from 1 to 4, and scores range from 31 to 124. The scale has a composite Cronbach’s α of 0.90, and a five dimensional Cronbach’s α ranging from 0.65 to 0.87. The scale ask questions such as "Whether you have professional promotion and development opportunities in current environment", and a higher C-NPES score shows higher recognition of the nurse’s roles and a more positive perception of the working environment. The *Chinese Worker Organizational Commitment Questionnaire* is a 25-item scale, which evaluated nurse’s affective, norm, ideal, economy, and opportunity commitments. The items are scored from 1 to 4, and total scores ranges from 25 to 100. It asks question such as "I will not leave the current hospital in any cases" with the answer "totally disagree" marked as 1 point and "totally agree" marked as 4. Higher scores showing higher organizational commitment. The test-retest reliability of the scale was 0.87, and the Cronbach’ α was 0.67 [25]. The *Nurse Career Commitment Questionnaire* is a 20-item scale, which included five dimensions, namely, emotional, norm, emotional cost, economic cost, and opportunity commitments. The questionnaire include questions such as "Many colleagues around want to leave the nursing field" and the answer "totally disagree" marked as 1 point and "totally agree" marked as 4. The score ranges from 20 to 80, and higher scores show higher career commitment. The Cronbach’s α is 0.91 [26]. The *Social Support Rating Scale* (SSRS) is a scale comprising 10 items which evaluate objective support, managerial support, and availability of social support, and include question such as "How many close friends do you have?". The total score ranges from 12 to 63, and higher scores show higher levels of social support. The Cronbach’s α of the scale is estimate at 0.92, and the coefficient of internal consistency of the three dimensions ranged between 0.89–0.94 [27]. The *Psychological Stress Scale* (PSS) evaluates the stress levels and includes 50 questions such as "I don't have a fixed sleeping time and I can't sleep well". The answers "always", "frequently", "sometimes", "seldom", and "never" will be marked 4, 3, 2, 1, and 0, respectively. Total scores ranging from 0 to 200, with higher score means the participant suffered more stress [28].

The *Scale of Intent to Leave the Profession* was translated and revised by LI Dongrong and LI Jingyuan [29] to evaluate the nurses' intention to turnover. The scale consists of 6 single-choice questions asking directly the respondent's intension to turnover. Each response was scored 1, 2, 3, or 4. The high scores indicate a weak intention to leave the profession. For example, "Have you ever considered to resign?”, and the answers "frequently", "occasionally", "seldom", and "never" would be marked as 1, 2, 3, and 4, respectively. The score of 4 indicates least intention to leave. The Cronbach’s α was 0.77, and the content validity was 67.67%.

### Data analysis

All data were statistically analyzed using SPSS software, v. 18.0 (SPSS, version 18.0, Chicago, IL, USA). Measurement data were presented as mean ± Standard Deviation (SD) (in normal or approximately normal distributions). Count data are presented as frequencies (percentage). For count data, the chi-square test was employed for group comparisons. Two-tailed tests of mean differences were used and the 0.05 level was used as the criterion for determining statistical significance. Multiple factor logistic regressions were used to analyze the hierarchical impact factors for turnover intention. Additional sub-group analysis was performed to determine whether professional title, job responsibility and hospital level influenced the turnover pattern in different age group nurses. Multi-level modeling techniques, clustered into six hospitals, were used in the study.

## Results

### Characteristics of sample

**Fig. 2:**
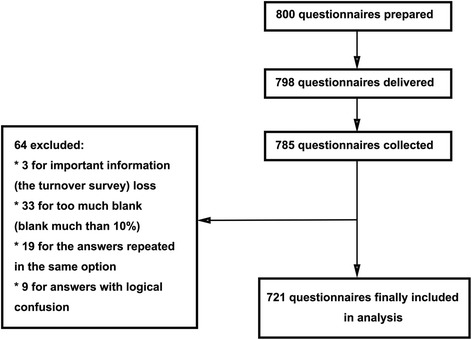
Flowchart of recruitment processes

A total of 785 of the 800 distributed questionnaires were collected (98%), of which 64 were excluded due to incomplete filling, inconsistencies, logical concerns, or missing information. A total of 721 (90%) valid questionnaires were available for statistical analysis (Fig. 2).

Study participants were aged between 20 and 55 years (29.85 ± 6.86), although most were between 20 and 29 years of age. Only 31 (4.29%) participants were male nurses, and nurses’ experience in the profession ranged from 1 to 34 years (8.40 ± 7.66) (Tables 1, 2 and 3). The average score for turnover intention was (15.00 ± 3.24) and the ratio of average score to the maximum score was 63.88%. For most, the intention of leaving the profession was mostly weak (62%) or very weak (19%), while only a minority had either a strong (19%) or very strong (<1%) intention to leave.

**Table 1:** Comparative characteristics of surveyed nurses, by turnover intention

Variable	Turnover intention (*n* = 721)							
	Strong/very strong (*n* = 138, 19%)		Weak (*n* = 447, 62%)		Very weak (*n* = 136, 19%)		Total	*P* value
	*n*	(%)	*n*	(%)	*n*	(%)		
Mean age, in years	30.09 ± 7.05		30.03 ± 7.17		29.00 ± 5.43			0.0791^a^
Mean experience, in years	8.49 ± 8.09		8.62 ± 7.93		7.58 ± 6.14			0.8792
Nationality								0.6016
Han	136	(19.24)	438	(61.95)	133	(18.81)	707	
Minority	2	(14.29)	9	(64.29)	3	(21.43)	14	
Marital status								0.6934
Unmarried	55	(19.37)	180	(63.38)	49	(17.25)	284	
Married	83	(19.17)	263	(60.74)	87	(20.09)	433	
Widowed	0	(0.00)	4	(100)	0	(0.00)	4	
Number of children								0.3588
None	3	(10.71)	22	(78.57)	3	(10.71)	28	
One child	72	(20.63)	214	(61.32)	63	(18.05)	349	
Two children	63	(18.75)	206	(61.31)	67	(19.94)	336	
Three children	0	(0.00)	5	(62.50)	3	(37.50)	8	
Primary education								0.8040
Nursing school	54	(18.75)	177	(61.46)	57	(19.79)	288	
Junior college	68	(18.48)	232	(63.04)	68	(18.48)	368	
College	16	(24.62)	38	(58.46)	11	(16.92)	65	
Highest level of education								0.3068
Nursing school	9	(31.03)	15	(51.72)	5	(17.24)	29	
Junior college	45	(16.67)	174	(64.44)	51	(18.89)	270	
College	82	(19.85)	255	(61.74)	76	(18.40)	413	
Professional title								0.0497
Resident nurse	73	(20.00)	225	(61.64)	67	(18.36)	365	
Nurse	43	(18.53)	136	(58.62)	53	(22.84)	232	
Fellow	16	(14.55)	79	(71.82)	15	(13.64)	110	
Nurse-in-chief	6	(42.86)	7	(50.00)	1	(7.14)	14	
Job responsibilities								0.0679
Nurse	87	(18.59)	299	(63.89)	82	(17.52)	468	
Teaching nurse	22	(15.94)	81	(58.70)	35	(25.36)	138	
Team leader	12	(27.27)	21	(47.73)	11	(25.00)	44	
Head nurse	17	(23.94)	46	(64.79)	8	(11.27)	71	
Authorized level								0.1797
Formal nurse	40	(20.51)	129	(66.15)	26	(13.33)	195	
Contract nurse	98	(18.63)	318	(60.46)	110	(20.91)	526	
Income								0.9590
< 322 US dollars	25	(16.78)	96	(64.43)	28	(18.79)	149	
322 ~ 483 US dollars	57	(17.33)	207	(62.92)	65	(19.76)	329	
484 ~ 644 US dollars	36	(21.82)	97	(58.79)	32	(19.39)	165	
≥ 645 US dollars	14	(17.95)	49	(62.82)	15	(19.23)	78	
Position								0.8656
Clinical work	125	(19.14)	408	(62.48)	120	(18.38)	653	
Administrative work	2	(33.33)	3	(50.00)	1	(16.67)	6	
Medical-technical	1	(12.50)	7	(87.50)	0		8	
Assistant	3	(18.75)	8	(50.00)	5	(31.25)	16	
Others	6	(23.08)	15	(57.69)	5	(19.23)	26	

^a^Likelihood ratio of the chi-square value

**Table 2:** Chi-square tests among groups between organization details of the participants

Variable	Turnover intention (*n* = 721)							
	Strong/very strong (*n* = 138, 19%)		Weak (*n* = 447, 62%)		Very weak (*n* = 136,19%)		Total	*P* value
	*n*	(%)	*n*	(%)	*n*	(%)		
Department								0.3114^b^
Surgery	44	(16.99)	166	(64.09)	49	(18.92)	259	
Medicine	46	(22.89)	116	(57.71)	39	(19.40)	201	
Emergency	17	(23.61)	43	(59.72)	12	(16.67)	72	
Operating room	2	(5.56)	25	(69.44)	9	(25.00)	36	
Outpatient	2	(8.70)	14	(60.87)	7	(30.43)	23	
Pediatric	9	(26.47)	20	(58.82)	5	(14.71)	34	
Others	18	(18.75)	64	(66.67)	14	(14.58)	96	
Hospital level								0.0428^a^
Tertiary hospital	125	(20.49)	376	(61.64)	109	(17.87)	610	
Secondary hospital	13	(11.71)	71	(63.96)	27	(24.32)	111	
Overtime work	1.96 ± 3.31		1.87 ± 2.94		1.43 ± 2.53			0.4283
Ratio of nurses and beds	0.65 ± 0.60		0.75 ± 0.88		0.73 ± 0.85			0.8397
Hospital location								
Urban	128	(19.25)	409	(61.50)	128	(19.25)	665	
Suburban	6	(23.08)	15	(57.69)	5	(19.23)	26	
County	4	(14.81)	20	(74.07)	3	(11.11)	27	
Socioeconomic status of the community								0.2881
Advanced	49	(22.79)	122	(56.74)	44	(20.47)	215	
Average	74	(17.62)	265	(63.10)	81	(19.29)	420	
Underdeveloped	13	(17.33)	52	(69.33)	10	(13.33)	75	

^a^Likelihood ratio of the chi-square value

^b^Rao-Scott chi-square value, stratified according to hospital and the level of the hospital

**Table 3:** Chi-square tests among groups between social/organizational measures

Variable	Turnover intention (*n* = 721)						
	Strong (*n* = 138, 19%)		Weak (*n* = 447, 62%)		Very weak (*n* = 136,19%)		Total
	*n*	(%)	*n*	(%)	*n*	(%)	
Prestige of working environment n (%), *P* = 0.7879							
Very low	0		0		0		0
Low	2	(16.67)	7	(58.33)	3	(25.00)	12
High	98	(19.80)	303	(61.21)	94	(18.99)	495
Very high	38	(17.76)	137	(64.02)	39	(18.22)	214
Organizational commitment n (%), *P* = 0.2991							
Very low	0		0		0		0
Low	12	(30.00)	20	(50.00)	8	(20.00)	40
High	109	(19.12)	357	(62.63)	104	(18.25)	570
Very high	17	(15.32)	70	(63.06)	24	(21.62)	111
Career commitment n (%), *P* = 0.7415^a^							
Very low	0		0		0		0
Low	3	(8.33)	28	(77.78)	5	(13.89)	36
High	107	(19.42)	340	(61.71)	104	(18.87)	551
Very high	22	(19.13)	69	(60.00)	24	(20.87)	115
Social support n (%), *P* = 0.8423							
Very low	9	(28.13)	18	(56.25)	5	(15.63)	32
Low	60	(17.70)	214	(63.13)	65	(19.17)	339
High	63	(19.50)	199	(61.61)	61	(18.89)	323
Very high	6	(22.22)	15	(55.56)	6	(22.22)	27
Work stress n (%), *P* < 0.0001							
No stress (0 ~ 59)	31	(11.44)	157	(57.93)	83	(30.63)	271
Low (60–70)	28	(24.78)	67	(59.29)	18	(15.93)	113
Medium (71–81)	20	(16.81)	83	(69.75)	16	(13.45)	119
High (82–92)	14	(17.07)	58	(70.73)	10	(12.20)	82
Very high (93–200)	45	(33.09)	82	(60.29)	9	(6.62)	136

^a^Likelihood ratio of the chi-square value

Table 4 presents the multiple factor logistic regression analysis results. The group with the weakest intention to leave the profession served as controls, and the OR was compared between the groups with weak and strong turnover intentions. Of the influential factors, age, work pressure, job duty and career commitment had the strongest impact on turnover intention (*P* < 0.01). Head nurses significantly have stronger turnover intention than the nurses without any positions (*P* < 0.01), as well as those having lower career commitment (*P* < 0.01). Nurses turnover intentions statistically varies by different grades of hospital (*P* <0.05) and nurses in tertiary hospitals were shown to have stronger turnover intention (20.49%) than those employed by secondary hospitals (1.71%).

**Table 4:** Multiple factor logistic regression of hierarchical impact factors

Characteristic	Turnover intention (weak)				Turnover intention (strong)			
	Adjusted OR	95% CI		*P*	Adjusted OR	95% CI		*P*
Age group								
20–24	Ref				Ref			
25–29	0.674	0.407	1.116	0.0007	0.879	0.442	1.749	<.0001
30–39	1.165	0.793	1.712	0.9662	0.895	0.456	1.759	0.6976
≥ 40	2.253	1.026	4.950	0.0307	1.119	0.314	3.986	0.8191
Job duty								
Nurse	Ref				Ref			
Teaching nurse	0.636	0.407	0.995	0.5363	0.638	0.466	0.874	0.0334
Team leader	0.395	0.237	0.659	0.0016	1.128	0.327	3.890	0.9863
Head nurse	1.348	0.740	2.455	0.0118	2.189	1.137	4.211	<.0001
Career commitment								
Low	Ref				Ref			
Very high	1.148	0.912	1.445	<.0001	1.185	0.919	1.528	<.0001
High	0.435	0.273	0.693	<.0001	<0.001	<0.001	<0.001	<.0001
Psychological stress scale	1.026	1.012	1.041	0.0004	1.035	1.018	1.052	<.0001

Nurses were divided into 20–24, 25–29, 30–39, and 40–49 age groups, and professional title, job responsibility and hospital level were set as variables that may influence the turnover pattern in different age groups. As revealed by multiple linear regression analysis, significant differences were only found in 30–39 age group (Table 5), while not in other age groups. The turnover intention was significantly (*P* < 0.05) higher in the team leader in the age group 30–39 positioned in the secondary hospital.

**Table 5:** Mutiple linear regression ananlysis of 30–39 age subgroup

Variable	β	t	*P*
Professional title			
Nurse-in-chief	Ref		
Resident nurse	1.096	0.451	0.653
Nurse	0.826	0.353	0.724
Fellow	0.797	0.341	0.733
Job responsibility			
Head nurse	Ref		
Nurse	−0.412	−0.567	0.571
Teaching nurse	0.621	0.831	0.407
Team leader	1.838	2.179	0.031
Hospital level			
Tertiary	Ref		
Secondary	3.029	3.781	<0.0001

## Discussion

The study identified work stress associated with age, duties, and career commitment as the common predictors of the intent to leave. The factors contributing to the work stress were highly specific to the demographically different social behavior in China as compared to the rest of world. As seen, the pediatrics department was affected by the highest turnover rate associated with work stress induced by overly concerned parents. Moreover, the income level was not a contributory factor, as an observation unique to the nurses’ in the Shaanxi Province of China. The intent to leave was strongest among the head nurses.

Nursing shortages are a worldwide concern and resignation of currently employed nurses leaving the profession serves as a major cause for this issue [2, 30]. It has been reported that between 1996 and 2000, work dissatisfaction led 28% of US registered nurses quit their jobs for non-nursing professions [31]. In Hong Kong Public Hospital, the overall nursing turnover rate is 4.5% and can exceed 5% in emergency wards [7]. A high turnover rate of nurse not only result in a blunt shortage of nurses, but also indirectly increase the workload, work stress and job burnout of remaining nurses which is itself associated with turnover intention, creating a vicious cycle. Interventions aimed at reducing turnover rates can, ultimately, increase job satisfaction and effectively alleviate nursing shortages. With the increasing population, China is facing a severe shortage in the nurse recruitment accompanied by a high nurse turnover rate. The current data on nurse turnover in China is limited to densely populated regions. In a nurse turnover bibliometric analysis spanning research articles from 2000 to 2015, Lyu et al. identified 72.8% of the studies from Beijing, Shandong, Shanghai, Guangdong, Heilongjiang, Jiangsu, Hunan, Zhejiang, Hubei, and Liaoning provinces [32]. A recent cross-sectional study in 10 European countries have highlighted the importance of country-to-country and region-to-region assessment of factors influencing the intent to leave [33]. Therefore, there is a need to amass the factors affecting nurses turnover in different provinces of China.

In this study, we surveyed a large sample of nurses employed at several hospitals in the Province. Our study, which is in agreement with previously published studies, showed that 19% and <1% of the nurses have a strong or a very strong turnover intention, respectively [34, 35]. Stratified multivariate logistic regression analysis showed that age, stress, duties and career commitment were main factors associated with turnover intention (*P* < 0.01). The sub-group analysis found the age 30–39 years as the pivotal predictors of intent to leave (*P* < 0.05), indicating that the nurses in high age groups but at low job responsibility level are more prone to leave the profession. Therefore, an age-based promotion can be considered as a corrective factor. Also, the nurses with higher job responsibility tend to retain the profession. Therefore, a performance-based increment in the professional title can be considered as an incentive to further withhold the nurses in the profession.

Younger nurses between the age of 25 to 29 years, which constituted the major age group of the current study, were in general more reliant to have a strong turnover intention. This is consistent with other results from domestic studies [36]. Younger or less experienced nurses may feel a greater amount of stress than their more experienced counterparts. They usually have shorter length of service, insufficient work experience, and knowledge that do not meet the requirements of clinical work, leading to increased worry about mistakes in their work, and greater stress [37]. Younger female nurses also juggle multiple societal roles as they face early married life and starting a new family. Many nurses quit their job because employment location and responsibilities interferes with their spouse’s professional requirements. Most nurses are female, and will be in need occasional maternity leave during and after pregnancy, and usually prefer to quit their jobs for their children and family. Rates of abortion among nurses reaches 39% [38], which is much higher than the incidence of general population, and serves to show that the work-family balance is a delicate one among Chinese nurses. The one-child policy in China may also have affected turnover rates among younger nurses who not only raise their own children, but are also the sole supporter for their aging parents. Our data demonstrates a slightly different situation is China, as compared to that in the USA and Europe where male nurses were associated with a greater intention to leave [39]. Holtom et al. theorizes that in an organizational setting, an increase in the tenure corresponds to an increase in the job embeddedness and job satisfaction, thereby corroborating with a decrease in the turnover [40]. Consequently, corrective measures to withhold the younger nurses for longer than 3 years through counseling, skill management, and learning could effectively assist to reduce the percentage of turnover.

Logistic regression analysis showed that stress is an important factor for turnover intention. The stress level among the nurses of Shaanxi Province is at staggering 62% Nurses with a "relatively high" intention to leave reported higher the stress levels also supporting a positive correlation between stress and turnover [41, 42]. A survey showed that overwork is the primary stress source for nurse [43] and the main reason for losing nursing staff [44, 45]. Professional nurses in emergency or critical care medicine is usually higher than those of other departments, which could lead to stronger turnover intention [45–47]. In our study, nurses in departments having larger workload exhibited a slightly stronger turnover intention (24% vs. 19%). We also observed similar findings for nurses working in pediatrics, where 26.5% of the nurses reported a stronger turnover intention. In China, pediatric nurses bear relatively higher stress due to tense relations between nurses and overprotective parents of children born under the one-child policy [48].

Another important stress inducer among Chinese nurses, is the effort-reward imbalance [48]. The high intensity of nursing work, with no great difference in their salaries, creates a strong sense of loss and higher intention of turnover [43]. The fact that most Chinese medical institutions follow a “physician-based, nurse-assisted” practice model, which leads to lack of initiative and independence in clinical activities, may also increase job dissatisfaction. Furthermore, younger nurses usually need to take care of a large number of non-nursing incumbent tasks, which may generate both confusion and dissatisfaction about their professional career [37]. An increased workload in clinical care, due to high hospitalization rates, reduces the young nurses’ ability to spare much time for their family, negatively affecting turnover intention in this age group. Another survey showed that although the salary of Chinese nurses is not high, they face very strong work intensity and family stress, which brings out the intention to depart from their professions [49].

The logistic regression analysis in this study also showed that head nurses, despite having more experience, a better position and a higher salary, have a significantly stronger turnover intention (*P* < 0.01). Even though the professional careers of these nurses are relatively successful, the turnover intentions do not show a decreasing tendency. It is possible that undertaking non-nursing management work, such as HR coordination, logistics support and so on, hinders the development in academic and management innovation. In addition, the appointment of head nurse remains conservative in China’s hospitals, as most head nurses are elected from the nurses with excellent clinical skills and rich nursing knowledge [50] irrespective of management abilities and without further training in administration. Thus, head nurses have very few standards upon which to guide their work [51] and may feel they are not qualified enough for their position.

Our results showed that career commitment is one of the factors that affect nurses’ turnover intention. Among nurses with a higher career commitment, the ratio of nurses having lower turnover intention is 80%, which is significantly different that those with lower career commitment (*P* < 0.01). There is a negative correlation between the level of career commitment and distribution of turnover intention, which is consistent with the relevant results from both domestic and foreign studies [52–54]. It is believed that the improvement of nurses' occupational commitment, by transition and innovation of nursing schools and medical institutions, is one of the most powerful methods against nursing shortage [52]. However, this method is inadequate for the complicated medical environment of China. In China, there is little career guidance, most nurses are unsure about their own career development and employers rarely have supporting policies. Most importantly, the majority of Chinese people still think that nursing is an occupation that does not require technical skills and, therefore, should be paid low [48]. Both the doctor-patient and nurse-patient relationships are tense in China, stemming from negative public opinion and sometimes resulting in violence. A survey for health care workers in Shenzhen, China, showed that >10% of medical workers have frequently experienced violence in the workplace [55]. These acts easily generate distrust among nurses towards their occupations and organizations, consequently resulting in their departure from the profession.

This study also analyzed nurses’ turnover intention in different grades of hospital, indicating that turnover intention in tertiary hospitals was higher than for secondary hospitals, which is consistent with the prior studies [56, 57]. The beds in tertiary hospitals are more frequently used, workload and work stress is comparatively higher, which induces turnover tendency [56] despite the fact nurses in tertiary hospitals prefer to keep their positions compared to the nurses in primary hospitals due to better income and career development [58]. However, we found no significant correlation between turnover intention and income of nurses in Shaanxi Province, which differed from previous result [48]. This is probably because nurses pay more attention to the balance of their work and reward, instead of the amount of their income. Another possible reason is that the qualification of most clinical nurses is graduation and their income is relatively higher than the people having same degree of other occupations. Also, the reason might be due to positive promotion of new policies such as excellent nursing service, multi-level management to solve the problem of unfair treatment on nurses’ salaries in the past. Hospitals with different grades are gradually promoting the new policy of “equal pay for equal work” to improve nurse satisfaction. Taken together, the present report substantiates the role of work stress and burnout as the major contributing factors towards the intent to leave affecting nurses’ turnover. However, the work stress of Chinese nurses is contributed by elements that differ significantly from other countries.

The current study is a cross-sectional study thus is with some limitations. Due to lack of manpower and material resources, complete randomization was not possible and we resorted to a stratified sampling strategy. Three investigators (who were not involved in the study design) were randomly assigned to the regions by random drawing. The assigned investigator was in charge of the whole study process of survey, such as liaising with the nursing department, supervising the data collection, thus minimizing the effects of a lack of universal randomization. This study did not assess the current situation of nursing turnover in proprietary hospitals or township hospitals in Shaanxi Province. Our future work will cover these hospitals by expanding the scope of research. We also did not explore the relationship between workplace violence and nurses’ turnover intention, and the issues regarding the poor medical environment and the tense nurse-patient relationship leading to increase nurse turnover in China still needs to be explored.

In conclusion, the current study emphasizes the reduction in work stress through effective learning, enhanced experience, and social cooperation to increase the career commitment required to withhold the turnover.

## Conclusion

We found that turnover intention among nurses are related to multiple factors included age, stress, job responsibilities, and career commitment. Stress, which stem from familial, societal and organizational issues, was the most important influencing factor leading to nurse turnover intention. From this research, it appears clear that higher levels of stress lead to greater intentions of turnover. The Chinese adaptations of the standardized questionnaires used in this study are reliable and valid and can be used to help healthcare managers identify areas of concern within their institution and take effective measures to prevent nurses from resignation.

## Abbreviations

PSS: Psychological Stress Scale
SD: Standard deviation
SSRS: Social Support Rating Scale
